# The First Meeting with Patients

**Published:** 1898-09

**Authors:** Charles A. Brackett

**Affiliations:** Newport, R. I.


### THE FIRST MEETING WITH PATIENTS.[1]

BY CHARLES A. BRACKETT, D.M.D., NEWPORT, R. I.

TWENTY-FIVE years ago, in front of this platform, my diploma as a graduate of this school was put in my hands by President Eliot. On that occasion it was my privilege to read my thesis to those who had assembled to do honor to the graduation of the class of 1873.

---

[1] A paper read before the Harvard Dental Alumni Association on June 27, 1898, on the occasion of their annual meeting.

Standing in the same spot, mindful that few quarter century anniversaries can come to any of us here, holding in esteemed and tender recollection numbers of most worthy workers in our calling then present, now passed on to their reward, and giving to all of you here to-day brotherly greeting, I am privileged to speak once more.

The subject assigned for to-day is an important one. The influence exerted by a dentist upon a patient in a first interview may be great and far-reaching, and may concern others than the two named. A first point to have in thought is the previous relations of the patient with other dentists. If one knows or has reason to suppose that a caller has been the patient of another practitioner in the near neighborhood, it is proper to seek to find out if the former relations may not be happily continued. If the caller express dissatisfaction with the treatment of any kind which has been received, I think that, in the majority of instances, a careful weighing of the statements and an inspection of the work will show that certainly or probably injustice is being done. I would not imply that all the perfection is on the side of the practitioner and all the imperfection on the part of the patient; but there are many things in the practice of dentistry of which none but a dentist can well judge. If we are able to restore to a questioner full confidence in a dentist who is worthy of it we have rendered the questioner a real service, we have saved our neighbor from injustice, and we have gained for ourselves the satisfaction of having done right from a worthy motive.

A few evenings ago I attended a meeting of a medical society, at which questions of ethics and of legislation regulating practice had been under discussion. After the adjournment it was my privilege to walk away with a lady who has been for many years a busy and successful practitioner of medicine. Referring to the discussion in which we had been participating, this lady said that if all doctors would conduct themselves with a high-minded honesty and nobility of purpose in their intercourse with patients and in their references to each other there would be little need for codes and laws. Too often there is a chain of circumstances like this: A patient is treated by a physician for a little time; dissatisfaction arises; physician number one is dismissed, and physician number two is called in. Physician number two is expected to express disapproval of what was done by number one, and number two meets the expectation and expresses the disapproval. I would not do injustice to physicians, but I think physicians themselves will admit that such things as these occur, and occur when they should not. Nor do I believe dentists are perfect; but it has been my good fortune to live and practise for twenty-five years in a community where there have been for different parts of the time something like a score of dentists, and I have no recollection of there ever having come to my knowledge in that time any unkind, insinuating, or disparaging remark made by any Newport dentist concerning the work of any other qualified dentist in the city.

It being settled that the caller is one who has just claims upon our service, it becomes incumbent on us to make that service as real and as acceptable as possible. At the outset we should seek to inspire in the patient confidence in us. Confidence is at the foundation—is the foundation—of all the worthy relations which individuals may have one with another. Go over in your minds all the interrelations and interdependencies of humanity and see how trust and confidence underlie and permeate the whole. Any one who ministers to any of the many ills to which flesh is heir comes into peculiarly intimate and confidential relations with those whom he serves,—confidential, because honor is involved; confidential, because, literally, confidence is involved. We need the patient's confidence in our professional capacity, and not only in our capacity but in our worthy intentions, in our honesty, in our sincerity, in our sympathy, in our faithfulness. If that confidence is not felt by the patient, there cannot be that cordial co-operation with us essential for the readiest attainment of the desired result, nor can we render the best service of which we are capable.

Now, how shall we inspire this confidence? Just simply by deserving it. In our neighborhood, in our office appointments, in our neatness, in our dress, in our manner, in our daily walk and conversation, in our personal self, and, most of all, in our mind and heart and soul, which is the real man, and which inspires and controls all the external expressions of the man, there should be the effort towards worthiness. Politeness is well, but politeness to be of any real value must be the expression, spontaneous and refined, of a kind heart, thoughtful for the comfort and happiness of others.

Blessed and blessing is the man or the woman who has tact, who does not see little mistakes and awkwardnesses in others, who can unobtrusively drive away bashfulness, aid the timid, encourage the shrinking, quiet the disturbed, make peace with the angry, and comfort the suffering. We should have high conceptions of our opportunities and responsibilities, of our privilege and duty to devote to useful and worthy ends the powers with which a kind Providence has endowed us.

When the new patient comes in we should show interest in the object of the visit and an earnest desire to render the service which the case requires. We should have patience in listening to what the patient may tell us to aid in the diagnosis, being at the same time mindful of using our own powers of discrimination. We should show deference to age, to peculiarities of temperament, to some extent even to "notions," if thereby the patient's interests are served. We should show kindness, patience, sympathy, and we should remember that in all of these things actions speak louder than words. Try not to impose upon the patient things especially repugnant unless they are really needed. Studiously avoid all careless and unnecessary hurts, and be firm and steady in making those hurts which are inevitable. Be ready to give good reasons for all that you do, and give warning in advance of anything that is likely to be particularly painful. Never deceive, and especially do not deceive a child, nor allow any one else in your presence to deceive a child in regard to an operation which you are to perform. If possible, so win its confidence that it will submit voluntarily to what must be done, but it is better to control its body by force than to warp its soul by deceit. Capacity to win the confidence of children is usually a measure of one's merit for the confidence of older persons. Of one of the contributors to this symposium, a lady, said, "Dr. C. quite won my heart. My little girl was ill in bed, and while she was ill she had a toothache; Dr. C. came to the house to comfort her, and from his kindness and goodness to my little daughter I knew that he must have children of his own."

Do not be pompous. No one gains in that way any respect worth having. Do not be familiar, "Familiarity breeds contempt." Do not be a "know-it-all," and do not try to "show off" in any way. Do not talk too much; do not talk too little; but better too little than too much. Do not ask irrelevant questions. Avoid everything of a gossipy nature. Do not talk of one patient to another patient. Try to say nothing that quoted to anybody anywhere might do harm, and remember the inaccuracy of usual spoken quotations.

Be cautious in diagnosis; seek to set aside as much as possible sources of error. Avoid too much positiveness. Be brave; be not too brave. Be very careful about definite promises. Guarantee nothing except faithful effort to accomplish the service needed. Impress the patient with the fact that the patient's interests and your interests are not antagonistic, but identical, and that your satisfaction in the matter must be consequent, in a measure, upon the patient's satisfaction.

Have some experience yourself in the dentist's chair occasionally, and be so mindful of that experience as to try and do as you would be done by. The man who would have friends must show himself friendly. Animals respond to the treatment which they receive, according to its nature. They shrink away from those who have abused them, who have played tricks upon them, who have been cross to them, while to those who have treated them fairly and who have shown them kindness, they return unmistakable evidences of confidence, and often of the most devoted affection. Human beings, as well as horses, dogs, and cats, have some powers of discrimination.

It is an honor to any dentist to have it truthfully said of him that his patients are his friends. There is reward and inspiration in that. In obituary notices of worthy men we often read that he who has passed away had enjoyed during his life the confidence of all those with whom he had business relations, or that he had enjoyed the confidence of the community in which he lived. It has always seemed to me that there was a particular appropriateness in the word enjoyed in that connection.

The expressions of confidence and trust in us, and of grateful appreciation of efforts which we have made to render service, as these expressions come to us day after day in our busy lives, are among the most pleasant rewards which we can have. They cheer the toil and lessen the fatigues of the present, encourage us to go on to do the work of the future, and inspire all our nobler nature to try to be worthy of the estimation in which we are held.

